# Self-Healing in Cementitious Materials—A Review

**DOI:** 10.3390/ma6062182

**Published:** 2013-05-27

**Authors:** Kim Van Tittelboom, Nele De Belie

**Affiliations:** Magnel Laboratory for Concrete Research, Department of Structural Engineering, Faculty of Engineering, Ghent University, Technologiepark Zwijnaarde 904, Ghent B-9052, Belgium E-Mail: kim.vantittelboom@ugent.be

**Keywords:** mortar, further hydration, encapsulation, polymers, bacteria, sustainability

## Abstract

Concrete is very sensitive to crack formation. As wide cracks endanger the durability, repair may be required. However, these repair works raise the life-cycle cost of concrete as they are labor intensive and because the structure becomes in disuse during repair. In 1994, C. Dry was the first who proposed the intentional introduction of self-healing properties in concrete. In the following years, several researchers started to investigate this topic. The goal of this review is to provide an in-depth comparison of the different self-healing approaches which are available today. Among these approaches, some are aimed at improving the natural mechanism of autogenous crack healing, while others are aimed at modifying concrete by embedding capsules with suitable healing agents so that cracks heal in a completely autonomous way after they appear. In this review, special attention is paid to the types of healing agents and capsules used. In addition, the various methodologies have been evaluated based on the trigger mechanism used and attention has been paid to the properties regained due to self-healing.

## 1. Introduction

Concrete is the most widely used construction material because of its high compressive strength, relatively low cost, *etc.* One adverse property of concrete is its sensitivity to crack formation as a consequence of its limited tensile strength. For that reason, concrete is mostly combined with steel reinforcement to carry the tensile loads. Although these rebars restrict the crack width, they are mostly not designed to completely prevent crack formation.

Cracks endanger the durability of concrete structures as aggressive liquids and gasses may penetrate into the matrix along these cracks and cause damage. Consequently, cracks may grow wider and the reinforcement may be exposed to the environment. Once the reinforcement starts to corrode, total collapse of the structure may occur. Therefore, it seems obvious that inspection, maintenance and repair of concrete cracks are all indispensable.

However, crack repair becomes difficult when cracks are not visible or accessible. Moreover, in Europe, costs related to repair works amount to half of the annual construction budget [1]. In addition to the direct costs, also the indirect costs due to loss in productivity and occurrence of traffic jams carry a severe economic penalty. Accordingly, self-healing of cracked concrete would be highly beneficial.

Self-healing is actually an old and well known phenomenon for concrete as it possesses some natural autogenous healing properties. Due to ongoing hydration of clinker minerals or carbonation of calcium hydroxide (Ca(OH)_2_), cracks may heal after some time. However, autogenous healing is limited to small cracks, is only effective when water is available and is difficult to control. Nonetheless, concrete may be modified to build in autonomous crack healing.

**Figure 1:**
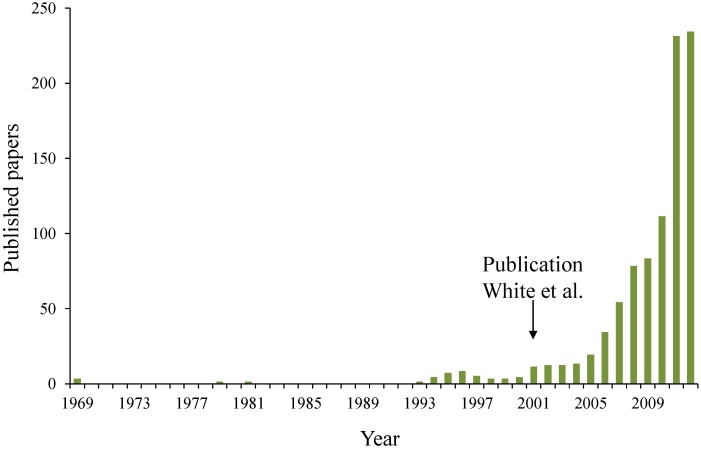
Evolution of the amount of papers published on self-healing materials (redrafted after slide shown by S. White during the International Conference on Self Healing Materials 2011 in Bath).

In 1969 [2], self-healing properties were for the first time built-in inside polymeric materials. In 1979 [3] and 1981 [4] publications about self-healing in thermoplastic and cross-linked systems appeared. In the 90’s, Dry started to work on self-healing concrete [5] and polymers [6]. Although, it was only in 2001 when White *et al.* [7] published their paper in Nature about self-healing in polymer based materials that the research on self-healing materials started to attract a lot of attention (Figure 1).

In this paper, the state-of-the-art on self-healing in cementitious materials is given. First, different approaches to self-healing are explained. Subsequently, the advantages and disadvantages of different types of healing agents and encapsulation techniques are discussed. This is followed by a discussion about the different trigger mechanisms which can be applied to activate self-healing. To conclude, the properties regained due to self-healing and the techniques used to quantify self-healing are considered.

## 2. Approaches to Self-Healing

Self-healing in cementitious materials can be classified broadly into three groups: intrinsic healing, capsule based healing and vascular healing, in accordance with approaches which originate from self-healing of polymers [8]. Each approach differs in the mechanism used to accomplish healing in the damaged region.

### 2.1. Intrinsic Self-Healing

Intrinsic self-healing materials exhibit self-healing properties due to the composition of the cementitious matrix. In this approach, healing relies on autogenous healing, improved autogenous healing or reaction of the polymeric substances inside polymer modified concrete.

#### 2.1.1. Autogenous Healing

One of the most widely studied mechanisms of intrinsic crack healing in cementitious materials is autogenous healing [9,10,11,12,13,14,15,16,17,18,19,20,21,22,23,24]. Autogenous crack healing can be mainly attributed to two mechanisms [10,22]: (1) hydration of unhydrated cement particles and (2) dissolution and subsequent carbonation of Ca(OH)_2_ [22,25]. Besides these two mechanisms, swelling of the matrix and blocking of the crack due to debris present in the ingress water or loose concrete particles resulting from cracking may also result in autogenous healing.

The overall contribution of these mechanisms remains a matter of debate. Apparently, the mechanism with the highest capacity to result in autogenous healing depends on the concrete age at the time of cracking. Due to its relatively high content of unhydrated cement particles, ongoing hydration is the main healing mechanism in young concrete. At a later age, calcium carbonate (CaCO_3_) precipitation becomes the major mechanism [9]. While different opinions exist about the main mechanism causing autogenous healing, researchers agree that for each mechanism, the presence of water is essential.

The maximum crack width that can be healed by autogenous healing was observed to differ substantially among reports made by various authors, *i.e.*, 5 to 10 µm [17,26], 100 µm [15], 200 µm [22], 205 µm [14] and 300 µm [27]. From the aforementioned studies, it follows that narrower cracks are more likely to be completely healed by autogenous healing. This was also proven by N. ter Heide *et al.* [28,29,30,31]. They investigated the effect of crack closure on the autogenous healing efficiency and concluded that improved healing was obtained when compressive forces were used to make both crack faces contact each other.

#### 2.1.2. Improved Autogenous Healing

**Figure 2:**
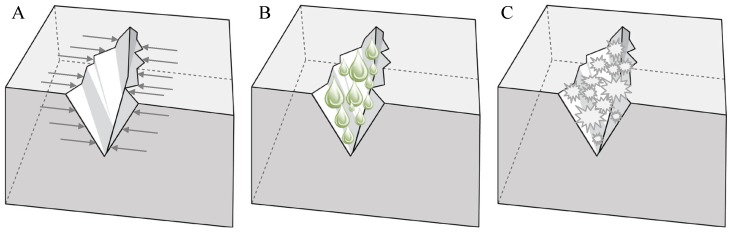
Intrinsic self-healing approaches. Improved autogenous healing by restriction of the crack width (**A**); water supply (**B**) or improved hydration and crystallization (**C**).

From what has been stated in the previous section, it becomes clear that autogenous healing is more effective when crack widths are restricted or crack closure can be acquired (Figure 2A). As water is always needed for autogenous healing to occur, the supply of water is another improvement factor (Figure 2B). To conclude, improving the possibility of ongoing hydration or crystallization, also promotes autogenous healing (Figure 2C).

##### Restriction of the Crack Width

Li and coworkers were the first to propose the use of fiber reinforced strain hardening engineered cementitious composite (ECC) to restrict the crack width and thus promote autogenous healing. In their early research, polyethylene (PE) fibers were used [32], while later on they used cheaper poly vinyl alcohol (PVA) fibers [24,33,34,35,36,37]. Typical of ECC is its high ductility and its unique crack pattern. Instead of one single crack with a large crack width, multiple cracks form in the ECC matrix and the maximum crack width remains below 60 µm.

The efficiency of steel cord (SC), polypropylene (PP), PE and PVA fibers was compared by Homma *et al.* [38] and Nishiwaki *et al.* [39]. While SC fibers showed minor crack closing efficiency as the steel started to corrode inside the crack, PVA fibers induced the highest healing efficiency. This was attributed to the fact that PVA fibers promoted the deposition of crystallization products, as hydroxyl groups, attached on the fiber structure, attracted calcium ions. In their later research, Mihashi *et al.* [40] combined the use of PVA fibers with embedded brittle tubes containing repair agent. The fluid repair agent chemically reacted with silica particles in the concrete matrix to form crystals. In this specific case, healing was more efficient when cracks became larger than 200 µm. For crack widths below 200 µm, the PVA fibers were not sufficiently pulled out so as to expose enough silica material to block the crack.

In the approach suggested by Sakai *et al.* [41] and Kuang and Ou [42,43], crack widths are not restricted at the moment cracks develop but at the time of unloading, cracks are closed due to the super elastic behavior of embedded shape memory alloys (SMA). At Cardiff University, they examined the feasibility of low-level post-tensioned cementitious materials using shrinkable polymer tendons [44,45,46,47,48]. In this case, cracked specimens were heated in an oven resulting in shrinkage of the embedded poly ethylene terephthalate (PET) tendons followed by crack closure.

##### Water Supply

Several researchers investigated the possibility to mix super absorbent polymers (SAP), also called hydrogels, into cementitious materials to provide additional water [49,50,51,52,53,54,55,56]. SAP are cross-linked polymers that can absorb a large amount of liquid and swell substantially to form a soft and insoluble gel. Their swelling capacity is highly dependent on the alkalinity and ionic concentration of the solution, so SAP particles show reduced swelling when mixed into fresh concrete. Upon cement hydration, SAP release the absorbed water and shrink, leaving behind small macro-pores. When cracks arise, they are likely to propagate through the pores and ingress of moisture via the crack causes the SAP to swell again. If the external fluid has a low ionic concentration, the SAP will swell more than in the concrete pore solution, hence expanding beyond the pore into the crack leading to a direct physical blocking effect. During dry periods, SAP release their water content again, stimulating autogenous healing.

As the initial swelling of SAP particles during concrete mixing causes the formation of pores and thus a reduction in strength, Lopez-tendero *et al.* [53] tried to modify the polymers so that they only swell at reduced pH level. During concrete mixing the pH is around 13, preventing swelling of the SAP. Xia [57] coated swollen SAP particles with paraffin wax, to prevent leaching of the water absorbed by the SAP particles during cement hydration, and hence, to prevent loss of the autogenous healing capacity.

In his study, Janssen [58] encapsulated pure water with paraffin. However, he noticed that 60% to 90% of the water was already lost over the first days after manufacturing the capsules as water migrated out through the capsule walls.

Another mechanism to promote the self-healing behavior, is the system proposed by Qian *et al.* [59]. They utilized nanoclay as an internal water reservoir in ECC and demonstrated that the water retaining capacity of nanoclay provided internal water for further hydration to occur.

##### Hydration and Crystallization

Other attempts to stimulate autogenous healing focus on the addition of agents which are able to promote the deposition of crystals inside the crack. Some researchers [26,31,37,60,61,62,63,64,65,66,67,68,69] replace part of the cement by fly ash or blast furnace slag which are respectively pozzolanic and latent hydraulic materials. As high amounts of these binders remain unhydrated even at the later age, autogenous healing due to ongoing hydration is promoted.

Other researchers used expansive additives to obtain improved healing. Sisomphon and Copuroglu [70] mixed in calcium sulfo aluminate based agents and crystalline admixtures. Upon ingress of water into the crack, ettringite crystals formed, which filled the crack. However, micro-cracks were noticed at the interfacial transition zone between the matrix and the aggregates, due to the expansive reaction. Therefore, they proposed encapsulation of the ettringite producing agents to reduce the risk of uncontrolled expansion and additional crack formation [71].

After their early developments [72,73,74,75], Ahn and Kishi concluded that crack healing can be obtained when replacing 10% of the cement content by a combination of expansive agents, geo-materials and chemical agents [76,77,78,79,80]. When water enters into the crack, the expansive agent expands, the geo-materials swell and the chemical agents cause precipitation of crystals leading to crack closure.

A disadvantage of applying particles which may further hydrate or crystallize is that their healing functionality is limited as the healing agent itself is consumed in the process. Jonkers [81] stated that it would be advantageous to use CaCO_3_ precipitating bacterial spores as they only mediate the healing process and are thus not converted. However, the bacteria will die when the cells become embedded by CaCO_3_ crystals and the bacterial activity will also come to an end when all nutrients are consumed. It can thus be concluded that even the bacterial approach will not allow an endless repetition of the healing process.

In their first attempt to create bio-based concrete, Jonkers and coworkers applied bacterial spores and nutrients into the fresh concrete mix [81,82,83]. When a crack appears in the matrix and water enters into the crack, both the spores and nutrients dissolve resulting in activation of the bacterial spores. Then, the bacteria initiate the production of CaCO_3_ crystals which deposit at the crack faces. The carbon dioxide (CO_2_), released due to metabolic conversion by the bacteria, reacts with Ca(OH)_2_ leached from the cementitious matrix to form additional CaCO_3_ crystals. Tests showed, however, that the spores only remained viable for a limited period as cells collapsed due to continuing cement hydration resulting in matrix pore diameters smaller than the 1 µm sized bacterial spores. In his later research (see Section 2.2.2), Jonkers protected the bacteria through encapsulation.

Although cells may be damaged due to continuing hydration, Li and Herbert [84] incorporated their bacterial spores without protection inside an ECC matrix. Up to now, they have only studied the influence of biomass and nutrients on the ECC properties. Viability tests still have to be performed.

#### 2.1.3. Healing in Polymer Modified Concrete

Abd-Emoaty [85] studied the autogenous healing behavior inside polymer modified concrete (PMC). PMC is made by the dispersion of organic polymers inside the mixing water of concrete. Upon cement hydration, coalescence of the polymers occurs resulting in a co-matrix of hydrated cement and polymer film throughout the concrete. They stated that healing in PMC occurred in the same way as in traditional concrete. However, healing occurred to a larger extent and was extended over a longer period compared to traditional concrete as more unhydrated cement was available in the matrix because the polymers enclosed the cement particles as a kind of membrane.

Katsuhata *et al.* [86] studied the self-healing efficiency of PMC containing epoxy resin without hardener. In this case, drops of epoxy with a hardened shell and a liquid core formed upon contact with alkalis and hydroxide ions [87]. Regain of the mechanical properties served as a proof that upon crack formation, the unhardened epoxy resin inside the drops filled the cracks and hardened upon contact with the alkalis or hydroxide ions.

In their research, Reddy and Liang [88] searched for a mechanism to make oil well cements watertight. They mentioned that the sealing efficiency of SAP particles depends on contacting the right type of fluid. Therefore, they preferred to use elastomers with low glass transition temperature, low melting point or low solid to liquid phase transition temperature or those which show cold flow, allowing fractured samples to self-heal without requiring contact with any type of fluid. A reduction of the water flow through cracked samples proved the efficiency of this approach.

Yuan *et al.* [89,90,91] proposed mixing ethylene vinyl acetate (EVA) copolymer particles into the matrix. Upon crack formation, the specimens were heated up to 150 °C, then the EVA particles melted and the adhesive flew into the crack, filling it and healing it.

### 2.2. Capsule Based Self-Healing

**Figure 3:**
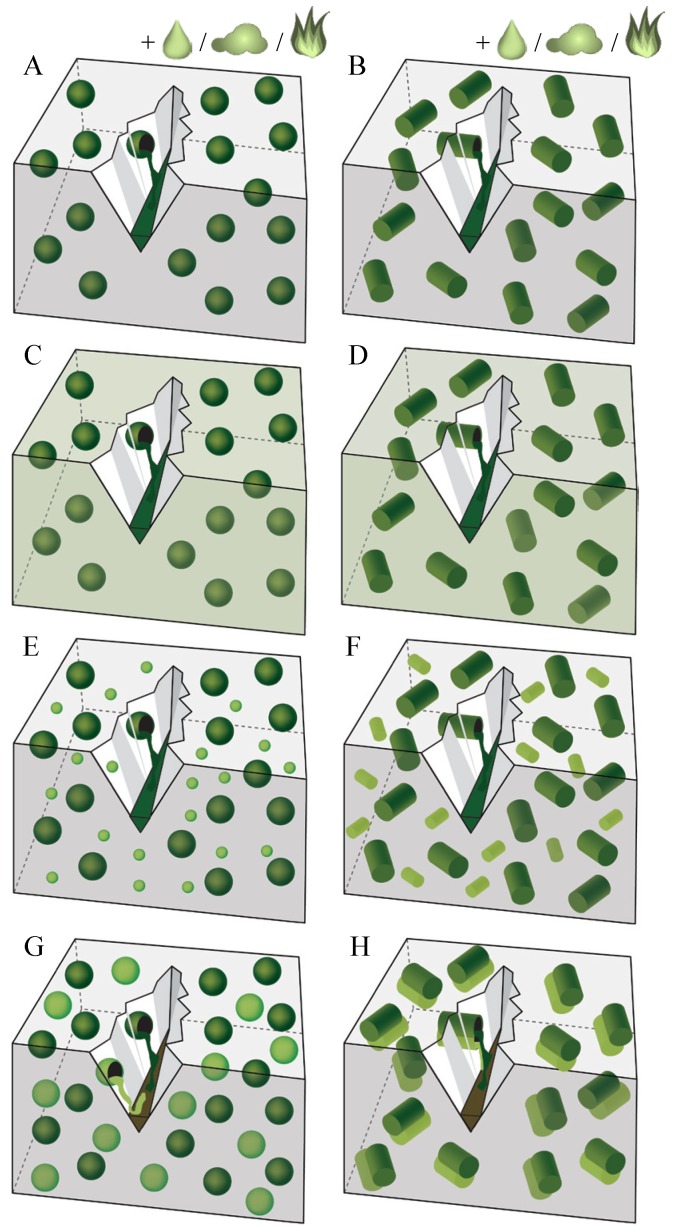
Capsule based self-healing approaches. Leakage of healing agent from the capsules into the crack due to gravitational and capillary forces. Reaction of spherical/cylindrical encapsulated agent (dark colored inclusions) upon contact with (**A**,**B**) moisture or air or due to heating; (**C**,**D**) the cementitious matrix; (**E**,**F**) a second component present in the matrix (small, light colored inclusions) or (**G**,**H**) a second component provided by additional capsules (big, light colored inclusions).

Capsule based self-healing materials sequester a healing agent inside discrete capsules. When the capsules are ruptured for example by damage, the self-healing mechanism is triggered through the release and reaction of the healing agent in the region of damage. While some agents react upon contact with moisture or air or due to heating (Figure 3A,B) or upon contact with the cementitious matrix itself (Figure 3C,D), other agents react when making contact with a second component which is present in the matrix (Figure 3E,F) or provided by additional capsules (Figure 3G,H). In the capsule based approach, capsules may have a spherical (Figure 3A,C,E,G) or cylindrical shape (Figure 3B,D,F,H).

#### 2.2.1. Reaction due to Moisture, Air or Heat

An example of the approach, depicted in Figure 3A, is the system explored by Cailleux and Pollet [1] where tung oil or Ca(OH)_2_ were encapsulated inside spherical microcapsules with a gelatin shell. When the encapsulated healing agent was mixed into a concrete repair mortar, it was shown that some capsules were destroyed during mixing, resulting in release of the encapsulated agent. Capsules which survived the mixing process only ruptured upon crack appearance. At that point, tung oil hardened upon contact with air. The other proposed healing agent, being Ca(OH)_2_, formed CaCO_3_ crystals upon reaction with CO_2_.

The mechanism, schematically presented in Figure 3B works along the same principle, although, the capsules have a cylindrical shape. Carolyn Dry applied this mechanism, when she embedded porous, cylindrical PP capsules filled with methyl methacrylate (MMA) and coated with wax in concrete. Upon crack appearance, the beams needed to be heated so that the wax coating melted and the healing agent was released, through the pores of the capsules. Due to the heat, MMA started curing inside the crack [5,92].

In her later research, Dry [92,93] made use of cylindrical glass capsules filled with cyanoacrylate (CA). Upon crack appearance, the brittle glass fibers broke, resulting in release of the CA which cured upon contact with air.

Joseph *et al.* [47,94], Van Tittelboom and De Belie [95] and Sun *et al.* [96] also used air curing CA encapsulated by cylindrical capsules. Joseph *et al.* noticed that almost no glue leaked out of the capsules upon crack formation. In his experimental setup, the capillary attractive force of the crack and the gravitational force on the fluid mass appeared to be insufficient to overcome the capillary resistive force of the cylindrical capsules and the negative pressure forces caused by the sealed ends.

To overcome the latter problem, Li *et al.* [32] combined the use of cylindrical glass capsules, containing CA, with the use of a high amount of PE fibers. The fibers restricted the crack width and thus increased the capillary forces to draw the healing agent out of the tubes. While some samples showed regain in stiffness upon reloading, the results for other specimens, from the same test series, were disappointing. Examination of cracked samples indicated that the healing agent had hardened inside the capsules before crack creation.

Instead of the very reactive CA, Pang and coworkers [97,98,99,100] used a one-component, epoxy healing agent. Similar to the previously mentioned studies, the healing agent was encapsulated by cylindrical glass capsules and cured upon contact with air. However, as this agent is less reactive, no hardening inside the tubes occurred before crack appearance.

To reduce the suction effect exerted by the sealed ends of the cylindrical capsules, de Rooij and coworkers [101,102] proposed encapsulating the healing agent inside coated hollow plant fibers. When cracks propagate in the concrete matrix, the fiber bundles tend to delaminate and the healing agent is released from the splintered fiber bundles into the damaged areas where it subsequently reacts.

#### 2.2.2. Reaction with the Cementitious Matrix

In the approaches presented by Figure 3C,D, premature hardening is excluded as the encapsulated healing agent can only react upon contact with a component which is part of the cementitious matrix.

After previous unsuccessful trials (see Section “Hydration and Crystallization”), Jonkers and coworkers [82,83,103,104,105,106], loaded expanded clay particles with both bacterial spores and calcium lactate (CaC_6_H_10_O_6_), which served as nutrient for the spores. As the clay particles prevented crushing of the spores, CaCO_3_ precipitation became possible. A disadvantage of this approach was that the expanded clay particles resulted in 50% decrease of the matrix compressive strength [103].

Huang and Ye [107] and Pelletier *et al.* [108,109] encapsulated a sodium silicate (Na_2_SiO_3_) solution into spherical capsules. As the capsules break and the Na_2_SiO_3_ solution is released, they react with the Ca(OH)_2_, naturally present in concrete, to form a calcium silicate hydrate (CSH) product that heals the crack.

#### 2.2.3. Reaction with Second Component Present in Matrix

The difference between the approaches described in the previous section and these represented by Figure 3E,F, is that the released healing agent does not react with the cementitious matrix itself but with an agent which has been added to the matrix to cause autonomous crack repair. An example is the mechanism proposed by Cailleux and Pollet [1] where bisphenol-F epoxy resin is encapsulated inside spherical microcapsules, embedded inside a concrete repair mortar. Inside the mortar matrix, a hardener was dispersed. When the epoxy resin is released from the capsules and contacts the hardener inside the matrix, a polymerization reaction is triggered.

Wang *et al.* [110] immobilized CaCO_3_ precipitating bacterial spores on diatomaceous earth to protect them from the high pH in concrete. In contrast to the bacterial strain (*Bacillus cohnii*) used by Jonkers *et al.* [83], the *Bacillus sphaericus* strain used by Wang *et al.* did not react with lactate but hydrolysed urea which was provided inside the cementitious matrix.

#### 2.2.4. Reaction with Second Component Provided by Additional Capsules

In the multi-capsule system (Figure 3G,H), two or more different types of capsules sequester separate components of the healing agent. Healing occurs upon rupture of both types of capsules and release of both components which then contact each other. An example of this approach, is the system proposed by Mihashi *et al.* [111]. They used spherical capsules with a urea formaldehyde formalin (UFF) shell containing a two component epoxy. The researchers, however, noticed that it was difficult for the two-component epoxy to harden, due to insufficient mixing of both components in the crack.

Feng *et al.* [112] also used spherical microcapsules with a urea formaldehyde (UF) shell filled with a two-component epoxy resin. However, these researchers modified the epoxy resin with a diluant chemical to adjust the viscosity, with the aim of obtaining superior mixing of both components. Although reaction of this epoxy resin may occur at room temperature upon contact with the curing agent, beneficial properties are obtained by thermal curing at 120 °C. Kaltzakorta and Erkizia [113] encapsulated a two component epoxy resin by silica microcapsules, instead of their polymeric counterparts. This research is still in its initial phase and up to now researchers have only proven the possibility to mix in this type of capsule in cement paste, so no statements can be made yet about the healing efficiency.

Yang *et al.* [114,115] used silica gel shell microcapsules filled with MMA monomer and triethylborane (TEB) serving as initiator. They showed that it was possible to mix the microcapsules into a mortar matrix and that contact of both components, after capsule breakage, resulted in bonding of the crack faces.

Van Tittelboom *et al.* [95,116,117] did not use spherical capsules but they encapsulated two-component healing agents by glass or ceramic cylindrical capsules which were connected two by two. Contact of both components upon breakage of the capsules was enhanced as they were positioned next to each other. Different types of healing agents such as epoxy resins, polyacrylates, polyurethane (PU) and MMA were tested on their efficiency. The best results were obtained with PU and MMA as polymerization of these agents is relatively insensitive to the mix ratio.

Wang *et al.* [118] investigated the possibility of improving the water tightness of the previously mentioned mechanism with encapsulated PU. She added a third tube and provided, next to both components of the PU, nutrients and CaCO_3_ precipitating bacteria. However, no additional improvement was noted. When silica gel instead of PU was embedded together with the nutrients and the bacteria an improved liquid-tightness was noted compared to encapsulation of only silica gel.

### 2.3. Vascular Based Self-Healing

**Figure 4:**
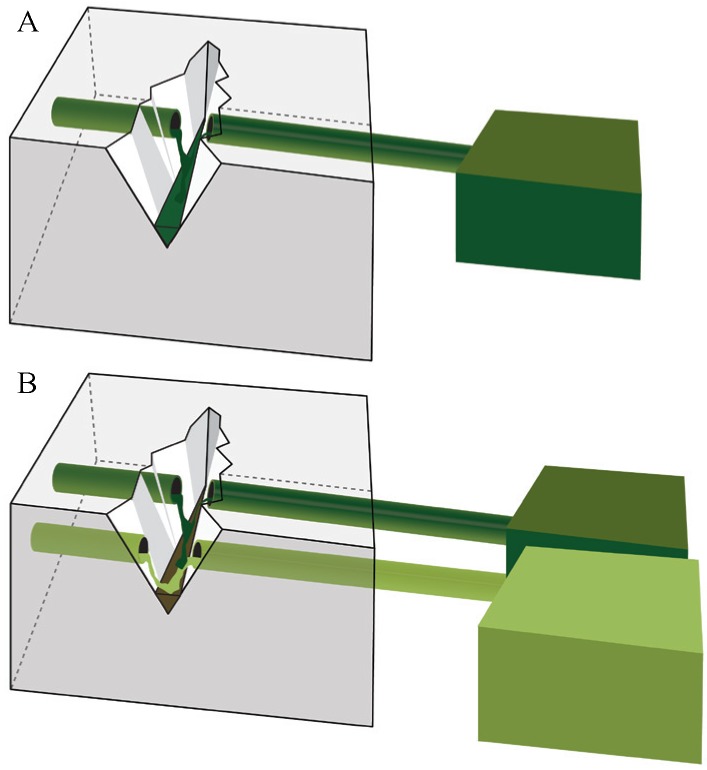
Vascular based self-healing approaches. Leakage of healing agent from the tank via the vascular into the crack due to gravitational and capillary forces and eventual (hydrostatic) pressure. One-channel (**A**) and multiple channel vascular system (**B**).

Vascular based self-healing materials sequester the healing agent in a network of hollow tubes which connect the interior and the exterior of the structure. When this approach is used in combination with a one-component healing agent, a one channel vascular system is applied (Figure 4A), while a multiple channel system is used in combination with a multi-component healing agent (Figure 4B).

#### 2.3.1. One Channel Vascular System

Joseph *et al.* [47,94,119,120] made use of an air-curing CA healing agent, provided by glass tubes. One end of the tubes was open to the atmosphere and curved to supply healing agent. When the tubes become depleted after cracking occurred, additional agent could be added via the open end to allow healing of wider cracks. Sun *et al.* [96] investigated the maximum healable crack width using this mechanism and concluded that the best results were obtained when the crack width remained smaller than 0.3 mm.

While generally beams are used to evaluate the self-healing efficiency, besides beams [121,122], Dry *et al.* tested other structural elements. They embedded glass tubes inside miniature concrete frames [93,123,124,125]. Tubes were each time connected to the exterior to fill them with the most suitable healing agent.

In the examples which have been mentioned up to now, additional agent needed to be supplied through human intervention when tubes became depleted. To overcome this, additional healing agent can be provided inside a tank which is connected to the outer end of the tubes. As tanks are placed at a higher level, gravitational forces will draw the agent into the glass tubes and subsequently the cracks. Mihashi *et al.* [111] used this technique in combination with an alkali silica solution as healing agent. Dry [5] connected a vacuum pump at one end of the tubes to suck the healing agent out of the tank and into the glass tubes.

Instead of providing the healing agent by long glass tubes, which are very prone to cracking during casting, Pareek and Oohira [126] provided holes inside the concrete. These were filled with epoxy healing agent by a syringe which was connected to the open end.

As the hollow core results in strength reduction, Sangadji and Schlangen [127,128] proposed using a hollow network which consisted of porous concrete. They cast porous concrete cylinders surrounded by a PVA film inside concrete beams. When the dense concrete matrix is cast around the porous part, the PVA film dissolves and thus exchange becomes possible between the inner and the outer layer. Damage can be detected by sensors, which switch on a pump that injects the epoxy healing agent available in a reservoir through the porous network to make it dense and heal the cracks.

Nishiwaki *et al.* [129,130,131,132,133] replaced the glass tubes by a pipe made of spiral twisted wire surrounded by an EVA film. Upon crack formation, a composite material consisting of a fiber reinforced glass matrix and a dispersed electro–conductive powder, which is provided below the film pipe and connected to a copper plate to make a thermal bridge, selectively heats the damaged part by electrification. This leads to melting of the surface of the organic pipe in the heated zone. Due to this, the epoxy healing agent is released and subsequently hardens inside the crack. In their latest work, Nishiwaki *et al.* [133] embedded their mechanism inside a layer of fiber reinforced concrete which was provided at the bottom of a beam.

Although, additional healing agent can be provided when the vascular based approach is used and thus even large cracks can be healed, the healing agent will leak out of the cracks when they become too wide. Therefore, Kuang and Ou [42,43] combined this approach with a mechanism to reduce the crack width. They included both SMA wires and continuous adhesive filled glass tubes. Upon damage the glass tubes break. Immediately after unloading, the deflection of the structural member is recovered due to the super elasticity of the SMA wire. At the same time the switch of the repairing vessel, containing healing agent, is turned on and the healing agent flows out of the broken, open fibers to fill and repair the cracks.

#### 2.3.2. Multiple Channel Vascular System

Mihashi *et al.* [111] embedded two glass pipes, which were connected to an external reservoir, into concrete beams. While one tube and connected reservoir were filled with one component of the epoxy glue, the other tube and reservoir were filled with the second component. Upon crack formation both pipes broke and both components were released resulting in a polymerization reaction. However, as both components did not mix well, the strength regain was almost similar to specimens without included healing agent.

In one of their studies, Dry and McMillan [134] used a three-part MMA based healing agent. By mixing two of the components, the system was reduced to a two-component system. Instead of using glass capillaries, rounded steel rods were cast in the concrete and pulled out after 24 h of curing. In this way tubular holes were left behind throughout the length of the samples which were then loaded with both components of the healing agent.

## 3. Efficiency of Different Types of Healing Agents 

In Table 1, an overview is given of healing agents used for application inside self-healing concrete. For each agent, the most relevant properties are mentioned. To suit as healing agent, several requirements need to be fulfilled, which are discussed in the following sections.

### 3.1. Rheological Properties

A very important parameter, is the viscosity of the healing agent. The viscosity should not be too high, in order to be able to flow out of the capsules and to fill the crack. Nevertheless, if the viscosity is too low, the agent could leak out of the crack or it could disappear due to absorption by the surrounding matrix.

**Table 1:** Overview of the healing agents which have been reported in the literature.

Agent	Number of components		Viscosity [mPas]	Way of curing	Curing time	Expansion		Strength [MPa]	References
	1	>2				Yes	No		
CA	√	–	<10	moist	seconds	–	√	20	[32,47,95,96]
Epoxy	√	–	–	moist, air, heat	60 °C, <100 min	–	√	–	[130]
	√	–	250–500	moist, air	–	–	√	22	[97]
	√	–	–	moist, air	–	–	√	25	[43]
	–	√	–	contact component	–	–	√	–	[111]
	–	√	–	contact component	±1 h	–	√	–	[1]
	–	√	200	contact component	–	–	√	17.6	[112]
	–	√	150	contact component	30 min	–	√	5.1	[95]
	–	√	80	contact component	30 min	–	√	4.2	[95]
	–	√	360	contact component	40 min	–	√	45	[95]
MMA	√	–	–	heat	–	–	√	–	[92]
	–	√	±1	contact component	30 min	–	√	50-75	[134]
	–	√	±1	contact component	–	–	√	–	[115]
	–	√	34	contact component	1 h	–	√	50	[117]
Silicone	√	–	–	air	–	–	√	–	[122]
Foam	√	–	–	–	–	√		–	[122]
PU	√	–	7200	moist	40–180 min	√		–	[95]
	–	√	600	contact component	50–300 s	√		–	[95,116]
Polyacrylate	–	√	7	contact component	40 s	–	√	–	[95]
Tung oil	√	–	–	air	–	–	√	–	[1]
Alkali silica solution	√	–	–	air	–	–	√	–	[111]
Ca(OH)_2_ solution	√	–	–	CO_2_ in air	–	–	√	–	[1]
Na_2_SiO_3_ solution	√	–	–	Ca(OH)_2_ matrix	–	–	√	–	[107,109]
Na_2_FPO_3_ solution	√	–	–	hydration and carbonation products	28 days	–	√	–	[135]
Ca(NO_2_)_2_ solution	√	–	–	matrix	–	–	√	–	[136]
PU + bacterial solution	–	√	600	contact component	–	√	–	–	[118]
Bacterial solution	√	–	–	water and O_2_	100 days	–	√	–	[137]
	–	√	–	water	–	–	√	–	[110]

Dry [138] concluded that the viscosity of the healing agent should be between 100 and 500 cps. Although CA has a much lower viscosity (<10 cps) it is frequently used in self-healing concrete. Researchers noticed that this agent filled up macro cracks and in addition, infiltrated into the region of micro cracks within the fracture process zone [47]. Here, it was experienced as an advantage that the agent was able to penetrate behind the crack faces as both the matrix around the crack and the crack itself were filled with hardened agent. This is characteristic for CA and is caused by its short curing time.

MMA is another example of a healing agent with a very low viscosity. As MMA does not cure as fast as CA (30 min *versus* a few seconds), MMA may be completely soaked up by the surrounding matrix and leave an empty crack behind. Conversely, as noticed by Dry and McMillan [134], the agent may leak out of the crack, which will also result in incomplete crack filling. Van Tittelboom *et al.* [117] increased the viscosity of MMA by the addition of poly methyl methacrylate (PMMA) as thickening agent, to retain the released agent inside the crack. This is more practical than the solution proposed by Huang and Ye [107]. They wrapped their specimens in foil during curing of the Na_2_SiO_3_ solution to prevent leakage.

Epoxy resins are mostly too viscous to penetrate into the crack. Although resins with a reasonable viscosity can be found on the market, Feng *et al.* [112] proposed adding a diluant chemical to adjust the viscosity of their epoxy resin.

### 3.2. Curing Conditions and Curing Time

As concrete structures may be in a wet condition at the moment of crack appearance, the healing agent should be able to react under wet circumstances. Some agents are not hindered by this fact as their reaction is initiated upon contact with moisture. When healing agents do not react upon contact with moisture, one-component agents react upon contact with air (tung oil [1]), CO_2_ in the air (Ca(OH)_2_ solution [1]), the matrix (Na_2_SiO_3_ solution [107,109] and calcium nitrite (Ca(NO_2_)_2_) solution [136]) or upon heating (epoxy [132] and MMA [92]). For these agents, the ability to cure in moist surroundings is an important parameter which needs further investigation.

Also the ability of two-component healing agents to react in a moist environment needs to be investigated. In addition, for two-component agents, the reaction kinetics upon inferior mixing of the components in combination with an improper mixing ratio should be a matter of concern. When Mihashi *et al.* [111] used epoxy for manual crack healing, selecting a proper mix ratio and mixing both components sufficiently, high strength regain was noticed upon reloading while this was not observed when both agents were embedded inside separate capsules and mixing depended on the outflow into the crack. As curing of epoxy resins is mostly very dependent on the correct mix stoichiometry, two-component epoxy resins are not appropriate for use in self-healing concrete.

The agent should not cure too fast as it should be able to fill the crack completely. But, on the other hand, quick setting adhesives, which are able to repair cracks almost instantly, may prevent further crack growth and may be beneficial in rapid cyclic loading conditions.

As a result of the short curing time of CA, Joseph *et al.* [47] already noticed regain in mechanical properties during crack creation. In their experiments, migration of glue in the cracked area below and above the capsules was noticed. Moreover, additional release of CA was noticed upon reloading. This let the authors conclude that despite the rapid curing abilities of CA when deposited in thin layers, the curing rate is significantly lower when released in larger volumes.

When other polymeric based agents such as epoxy or MMA are encapsulated, healing takes somewhat longer. Tran Diep [100] only noted complete curing of his epoxy resin seven days after crack formation, however, Nishiwaki *et al.* [130] were able to reduce the healing time of their epoxy resin to 100 min by heating at 60 °C. Nevertheless, when the latter agents are encapsulated, healing occurs still much faster than the time needed for an alkali silica or bacterial solution to react. In addition, the bacterial spores will only become active a few days after water entrance as the spores need some time to germinate. Wiktor and Jonkers [105] noticed that bacterial crack healing started after 20 days immersion in water and continued until 100 days of water immersion was reached.

### 3.3. Sealing Ability

Upon crack appearance, an additional volume is created. When the healing agent is released from the capsules to fill this volume, an empty space remains inside the capsules. Therefore, it would be beneficial if the healing agent expanded upon polymerization, so that a bigger crack space could be filled while only a small volume remain occupied during encapsulation.

The expansive reaction of the PU used by Van Tittelboom *et al.* [116] did not only fill the additional space but also acted as a driving force for the healing agent to come out of the capsules. Although an expansive reaction is required to seal the crack completely, care is needed that the expansive strain does not exceed the tensile strain capacity of concrete. Sisomphon and Copuroglu [70] noted numerous micro cracks due to expansion upon ettringite formation, which served as a repairing agent.

When temperature differences and cyclic loads cause the crack to grow wider or become more narrow, elastic behavior of the hardened agent is wanted. In order not to lose the bond between the repair agent and the cementitious matrix, and thus preserve the crack sealing ability, Letsch [139] stated that for manual crack repair, agents with a compressible volume need to be used. By mixing micro balloons containing compressible gasses into epoxy resins, he formed a new type of repair material fulfilling these requirements. Dry *et al.* [122] introduced a similar agent into their self-healing concrete, by embedding empty PP beads in encapsulated CA.

### 3.4. Mechanical Properties

The initial expanding action and the elastic behavior are both very important to guarantee the air and water tightness of the crack and prevent the occurrence of degradation. Regain in mechanical properties is of less importance, however, if the repaired crack is weaker than the surrounding concrete matrix, later cracks may arise at the same location where the healing agent is already exhausted. Therefore, it is important that the strength of the repair agent and the bond strength between the repair material and the concrete matrix are higher than the concrete strength.

While the concrete tensile strength is usually not higher than 5 MPa, the tensile strength of cured CA is around 20 MPa [47], the strength of PMMA ranges from 50 to 75 MPa [140] and for epoxy resins tensile stresses ranging from 5 to 45 MPa have been reported [43,97,112]. While the alkali–silica solution used by Mihashi *et al.* [111] has a strength which is less than the values noticed for polymeric based agents, the reported strength is still higher than that of concrete.

Further desired mechanical properties of the hardened repair material depend on the type of application. The release of stiffer adhesives allows the damaged structure to regain lateral stiffness. They transfer the stresses better but they will only allow a smaller margin of crack movement. More flexible resins may assist the damping characteristics of the structure, allowing a greater crack movement, but possessing less stress transfer capability.

Dry *et al.* [122,125] examined adhesives with low and high modulus of elasticity. Upon reloading of the specimens it was noticed that adhesives with higher modulus of elasticity such as CA can prevent reopening of the crack and transfer stresses to weaker sections of the member, while low modulus adhesives such as silicones and foams, allowed reopening of the crack but attempted to dissipate energy.

### 3.5. Stability over Time

As cracks may also appear in older concrete structures, the healing agent, which is the active component of the self-healing mechanism, should remain active, even after several years. One-component agents usually react upon contact with moisture or air or due to heating. Enclosure of air bubbles inside the capsules or intrusion of air through the capsule wall will result in early hardening of the agent. This was noticed by Li *et al.* [32] and Van Tittelboom and De Belie [95] who used air curing CA as healing agent. Also rise in temperature due to heating by sunlight may result in premature hardening of the healing agent inside the capsules.

In the vascular based approach, tubes can be loaded with healing agent after crack appearance, however, as the continuity of the tube may be disturbed when the agent hardens inside the broken part of the tube, the stability over time is also impaired. Joseph *et al.* [120,141] suggested the use of a three dimensional interconnected hollow network or the use of one dimensionally shaped tubes consisting of an outer brittle tube and an inner flexible, permeable tube to preserve its continuity.

To increase the stability over time, the use of a two-component healing agent can be preferable. As both components are embedded within separate capsules, no risk exists of premature hardening. Nevertheless, the reactivity of the individual components remains an important issue.

In the approach of Jonkers [103] and Wang *et al.* [110] the concern may exist as to whether the bacteria are still active after a long time. Therefore, Jonkers did not work with active bacterial cells but spores. Spores are dormant cells that can withstand mechanical and chemical stresses and remain viable for periods of over 50 years. From currently running viability experiments it has already been proven that after six months concrete incorporation no loss of viability is observed when spores are protected against crushing.

## 4. Suitable Encapsulation Techniques

In Table 2, an overview of different encapsulation techniques is given. In the following paragraphs, the most important demands for capsules are considered.

### 4.1. Survival of Mix Process and Influence on Workability

To facilitate the production of self-healing concrete, encapsulated healing agents are preferably added to the concrete mix during preparation. This implies that the capsules should be able to survive the mixing process. Not only due to the forces applied by the concrete mixer but also because of the impact forces of the aggregate particles, survival of the mix process is a big challenge. Moreover, addition of the capsules should not influence the workability of the fresh concrete too much.

It is obvious that tubular encapsulation systems cannot be applied during the mix process. Tubes need to be placed into the moulds beforehand, which results in additional handling and thus additional costs. Even at the time of concrete casting, care will be needed in order not to break the long brittle tubes. Therefore, the authors think that this approach is not suitable for practical application.

**Table 2:** Overview of the encapsulation techniques which have been reported in the literature (“–” means “not reported”, “x” means “not applicable”, “√” means “yes” and “/” means “no”).

Shell material			Content	Ø_i_	Ø_o_	Wall thickness	Length	Mixed in	References
				[µm]	[µm]	[µm]	[mm]		
Capsule based approach	Spherical	expanded clay	Na_2_FPO_3_	x	4000	x	x	√	[135]
		expanded clay	bacteria	x	1000–4000	x	x	√	[105]
		expanded clay	CaC_6_H_10_O_6_	x	1000–4000	x	x	√	[105]
		diatomaceous earth	bacteria	x	–	x	x	√	[110]
		gelatin	acrylic resin	–	125-297	–	x	–	[111]
		gelatin	epoxy	–	50	–	x	√	[1]
		gelatin	tung oil	–	50	–	x	√	[1]
		gelatin	Ca(OH)_2_	–	50	–	x	√	[1]
		wax	retarder agent	–	120	–	x	√	[142]
		paraffin	water	–	900	–	x	–	[58]
		cement + paraffin	SAP	–	–	–	x	–	[57]
		UF	epoxy	–	120	4	x	√	[112]
		UFF	epoxy	–	20–70	–	x	–	[111]
		PU	Na_2_SiO_3_	–	40–800	–	x	√	[109]
		silica gel	MMA	–	4.15	–	x	√	[115]
		silica gel	TEB	–	4.15	–	x	√	[115]
		silica	epoxy	–	–	–	x	√	[113]
		silica	Na_2_SiO_3_	–	5000	–	x	∕	[107]
	Cylindrical	glass	CA	800	1000	100	100	∕	[32]
		glass	CA	800	–	–	75	∕	[47]
		glass	CA	1500	–	–	75	∕	[47]
		glass	CA	3000	–	–	100	∕	[47]
		glass	epoxy	3000	5000	–	250	∕	[97]
		glass	epoxy	4000	6000	–	250	∕	[97]
		glass	epoxy	4000	7000	–	–	–	[97]
		glass	CA	3200	4000	400	200	∕	[96]
		glass	CA	–	100	–	63.5	√	[93]
		glass	CA	2000–3000	2200–3350	100	20–80	∕	[95]
		glass	epoxy	2000–3000	2200–3350	100	20–80	∕	[95]
		glass	polyacrylate	2000–3000	2200–3350	100	20–80	∕	[95]
		glass	PU	2000–3000	2200–3350	100	20–80	∕	[116]
		glass	bacteria	2000–3000	2200–3350	100	20–80	∕	[118]
		ceramics	PU	2500–3500	3000–4000	250	15–50	∕	[116]
		perspex	epoxy	–	–	–	–	∕	[97]
		plant fiber	–	–	40–188	–	–	–	[102]
		PP with wax	MMA	–	–	–	–	∕	[5]
Vascular based approach	Tubular	glass	alkali silica	800	2000	600	x	∕	[111]
		glass	epoxy	800	2000	600	x	∕	[111]
		glass	CA	3000	4000	500	x	∕	[47]
		glass	epoxy	4800	6000	600	x	∕	[42]
		glass	CA	3200	4000	400	x	∕	[96]
		glass	foam	1500	–	–	x	∕	[122]
		glass	epoxy	1500	–	–	X	/	[122]
		glass	silicon	1500	–	–	X	/	[122]
		glass	CA	1500	–	–	X	/	[122]
		spiral twisted wire with EVA	epoxy	2000	3400	700	x	∕	[130]
		porous concrete	epoxy	–	25000–35000	–	x	∕	[127]

For both spherical and cylindrical capsules, the possibility exists of mixing them in, however, spheres will more easily survive the mixing process and moreover, they will influence the workability of the matrix to a lower extent. In several of the studies listed in Table 2, spherical capsules were already successfully mixed in.

Among the cylindrical capsules listed in Table 2, plant fibers would have the most potential to survive the mixing process. In other approaches, brittle materials such as glass or ceramics have been used and these would probably not survive the mixing process. To withstand mixing, the wall thickness of the capsules can be increased. However, while this may prevent rupture upon concrete mixing, with a higher wall thickness, the fracture of the tube may not be timely to cause release of the healing agent and subsequent crack repair. This was experienced by Tran Diep *et al.* [97]: while glass tubes with a wall thickness of 2 mm were able to rupture upon crack formation, 3 mm thick tubes did not break. In their later research [98], glass tubes were coiled with a spiral wire and a 3.5 mm thick mortar layer. This did not yet allow mixing in of the capsules but it served as protection against premature damaging during casting.

Dry [123] proposed bundling cylindrical glass capsules by a water soluble glue to survive the mixing process. During mixing, the capsules become dispersed, as water in the concrete dissolves the glue. She proved that using this technique, cylindrical glass capsules were able to survive the mix process inside a truck mixer.

Dry [138] also proposed focusing further research on the development of capsules which are flexible at the moment of manufacturing and become more brittle upon hardening of the concrete mix.

The authors believe that the use of brittle encapsulation materials, which do not survive mixing, may be possible for precast concrete elements. Although, an additional step will be needed to place the capsules in the moulds, resulting in additional costs, capsules can be concentrated in the zones where they will be needed so less capsules have to be added. This will not only lead to a decrease in cost, but also the mechanical properties of the matrix will be influenced to a lesser extent.

### 4.2. Influence on Mechanical Properties

Inclusion of hollow capsules filled with healing agent may influence the tensile and compressive strength of concrete. Moreover, after release of the healing agent, spherical, cylindrical or tubular holes remain in the structure. Therefore, capsule dimensions need to be small enough in order not to change the properties of the structure too much.

In their study, Feng *et al.* [112] used UF spherical microcapsules with a diameter of 120 µm and concluded that the concrete compressive strength remained unaffected by the presence of these capsules. Also Pelletier *et al.* [109] noted that the compressive strength remained unaffected when spherical PU microcapsules were embedded. In general, spherical capsules will have less influence on the mechanical properties as their shape reduces the stress concentrations around the void left from empty capsules [47].

### 4.3. Compatibility with Matrix and Healing Agent

Once the capsules survived the concrete mixing and casting process, it is important that they remain stable within the highly alkaline cementitious matrix and that the shell is not affected by the encapsulated material. Therefore, the use of inert encapsulation materials, such as glass, may be beneficial. Although glass is used frequently as encapsulation material, it should be noted that this may induce alkali-silica reaction when a high amount of alkalis are present in the cementitious matrix. This drawback is avoided when inert ceramic capsules are used as proposed by van Tittelboom *et al.* [116].

In their study, Tran Diep *et al.* [97] compared the efficiency of cylindrical capsules made from glass and perspex (PMMA). Although perspex is stronger and more ductile than glass, this material was not suitable as it reacted with the healing agent. Other organic materials such as PP, PU, UF, EVA and gelatin gave good results when used as encapsulation material [1,5,92,109,111,112,129,130]. However, it is not known whether these researchers paid special attention to the compatibility as in the research of Tran Diep *et al.*

Kaltzakorta and Erkizia [113] used silica capsules instead of their polymeric counterparts, since the chemical nature of these microcapsules makes them more likely to be compatible with the cementitious matrix and may result in a better interface.

### 4.4. Probability of Crack Going through

When using encapsulated healing agents, the active component is not ubiquitous in the matrix but concentrated at specific locations. To increase the reliability of the system, enough capsules need to be included, so that the probability of a crack crossing the capsule and fracturing it, is sufficient. On the other hand, increasing the amount of capsules, will decrease the matrix intrinsic properties and increase the cost. Therefore, a compromise has to be sought for the amount of added capsules.

In general, it can be stated that cracks of a bended beam will always move towards the location of an included tubular self-healing system. If the crack length becomes higher than the concrete cover on the tube, they will certainly cross each other.

In the case of embedded spherical or cylindrical capsules, there is not a 100% chance of the crack going through. Zemskov *et al.* [143] developed two analytical models to determine the probability of a crack hitting spherical capsules. These models should allow the estimation of the needed combination of crack width, capsule size and intercapsule distance to obtain a certain self-healing efficiency. However, if the capsules form weak points within the matrix, they will attract the crack and increase the probability of rupture. Due to the higher surface to volume ratio of cylindrical capsules compared to spherical ones, their fracture probability is higher.

Moreover, as the ends of cylindrical capsules are somewhat anchored within the matrix, the chance of fiber pull out instead of fracture is much lower compared to that for spherical capsules. For spherical capsules, the bond strength between the outer capsule wall and the matrix needs to be stronger than the strength of the capsule to make the crack go through it instead of going around [111]. Some of the UF spherical capsules used by Feng *et al.* [112] broke straight away, but a small number of them were simply pulled out.

### 4.5. Release Efficiency

Once the capsules are broken, it is important that they easily release the healing agent. When tubular encapsulation systems are used, release can be improved through intervention from the outside of the structure. For example, the healing agent can be drawn through the tube and into the crack by a vacuum pump [92]. Placing the container which holds additional healing agent, somewhat above the structure, results in hydrostatic pressure [92,111], or moderate pressure can be exerted by a balloon [144].

In the case when separate volumes of healing agent are provided within the matrix, spherical capsules result in a more controlled and enhanced release of the healing agent compared to cylindrical capsules. For cylindrical shaped capsules, the release upon cracking is inferior because of the suction effects exerted by the closed ends. To make sure that the attractive forces inside the crack are higher than the capillary forces which keep the healing agent in the capsule, the crack width of the matrix should be limited to less than the inner diameter of the capsules. However, making use of encapsulation materials which are somewhat repellent to the encapsulated healing agent may allow a reduction in the diameter of the cylindrical capsules.

### 4.6. Healable Crack Volume

Rule *et al.* [145] stated that autonomic delivery of healing agent gives the same healing performance as when a similar amount of healing agent is delivered manually. However, when the crack volume exceeds the amount of healing agent available in the capsules, less successful healing is achieved.

The type of encapsulation technique used will influence the maximum allowable crack width which can be healed. Tubular capsules, connected to the outside, allow additional healing agent to be provided or to equip the system with an external reservoir, so that large cracks can be healed. A disadvantage of these tubing systems is that excessive healing agent may be released, resulting in aesthetical problems.

When spherical or cylindrical capsules are embedded, no additional supply is possible, so therefore, capsules need to be large enough to be able to transport a sufficient amount of healing agent to the crack. In the case of spherical capsules, the used capsule diameters reported in the literature range from 5 µm up to 5 mm. For cylindrical capsules the diameters range from 0.8 mm up to 5 mm.

**Figure 5:**
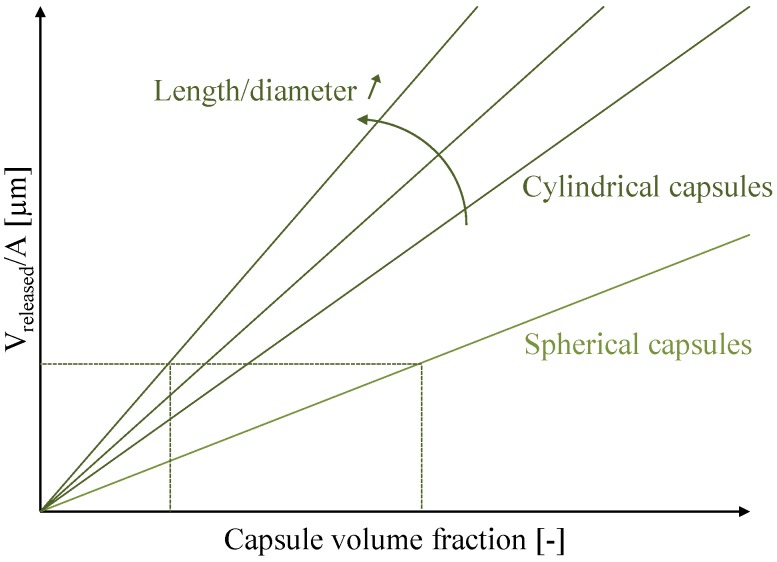
Released volume of healing agent (V_released_) per crack area (**A**) as a function of the capsule concentration for spherical and cylindrical capsules with a different length to diameter ratio. The horizontal and vertical crossing lines indicate the magnitude of the effect when changing from spheres to cylindrical capsules with a high length to diameter ratio (redrafted after [146]).

Spherical shaped capsules are rapidly exhausted when the crack width increases [111]. In their study, Feng *et al.* [112] noted that some cracks were filled with epoxy released from the spherical capsules while other cracks were not completely filled due to exhaustion of the capsules. Mookhoek [146] stated that for the same capsule volume fraction, a larger amount of healing agent could be delivered to the crack surface by cylindrical capsules and thus larger crack volumes can be healed. Besides, Mookhoeks’s model proved that increasing the length to diameter ratio results in an additional released volume per crack area (Figure 5). This can be converted into a decrease of the volume fraction of capsules, to obtain a similar specific healing potential. Such decrease lowers the effect of the liquid inclusions on the intrinsic material properties.

Also, the spatial orientation of the cylindrically shaped capsules inside the material is a parameter which can influence the release efficiency. Capsules oriented perpendicular to the crack plane result in a higher release efficiency compared to randomly oriented ones, due to the increase in fracture probability. This efficiency further decreases when capsules are oriented, aligned with the crack faces. In the latter case, the release efficiency is even lower than for spherical capsules.

## 5. Mechanisms to Trigger the Autonomous Healing Action

To obtain autonomous crack healing, a mechanism is needed to trigger the healing action. The self-healing approaches, which have been reported in the literature up to now, are triggered by ingress of liquids or gasses, exertion of heat or by crack formation (Table 3). Below, for each trigger mechanism, the main advantages and disadvantages are discussed.

**Table 3:** Overview of the mechanisms which might trigger self-healing of cracks.

Trigger		Result
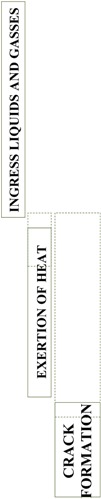	water	Autogenous healing | further hydration
	water + CO_2_	Autogenous healing | CaCO_3_ precipitation
	water	Expansion, swelling and precipitation of additives
	water	Swelling of SAP and autogenous healing [49,52,147]
	high RH	Swelling of SAP and autogenous healing [56]
	chloride solution	Coating around porous PP tube degrades and Ca(NO_2_)_2_ leaches through pores
	water + O_2_	Activation of spores and bacterial CaCO_3_ precipitation [103]
	water	Bacterial CaCO_3_ precipitation [110]
	CO_2_	Degradation of coating around expanded clay particles and release of Na_2_PFO_3_
	External	
	90 °C (+water)	Crack closure by SMA (PET) (followed by autogenous healing)
	100 °C	Melting of wax coating around porous PP capsules and release of MMA
	150 °C	Melting of EVA particles
	Internal	Melting of paraffin coating and release of hydration retarder agent
	48 °C	Melting of EVA film around Spiral wire and release of epoxy
	93 °C	
	(+water)	Crack closure by SMA (followed by autogenous healing)
		Capsule breakage and release of healing agent
		Delamination of plant fibers and release of healing agent
		Actuation of pump and injection of healing agent into porous concrete layer

### 5.1. Ingress of Liquids and Gasses

An advantage is that for this trigger mechanism the healing agents themselves are mostly dispersed in the matrix or contained within the matrix aggregates, so they can be mixed in during concrete manufacturing.

A disadvantage is that as long as the required agent does not intrude into the crack, healing is not activated. In the period between formation of damage and activation of healing, degradation of the concrete matrix can still occur. For example, SAP particles block the crack at the moment water enters, but do not prevent ingress of gasses during dry periods.

To qualify the efficiency of approaches using water ingress as trigger mechanism, samples are submerged in water or exposed to wet/dry cycles during the complete period of healing. However, in practice most structures will only be wetted due to rainfall and some may even never get wet. In the latter case, water triggered systems will not become active, except maybe for the systems containing SAP. It was noticed by Snoeck [54] that SAP particles already induce autogenous healing upon exposure to a high relative humidity as they are able to capture moisture from the air. More research is needed to find out whether the other healing mechanisms can also be triggered using more realistic conditions.

To activate the bacterial self-healing concrete proposed by Jonkers *et al.* [83], both water and O_2_ need to be present inside the crack. While water is sucked into even the tiniest micro cracks due to capillary action, the question may arise as to whether enough O_2_ will be present deep inside the cracks. Up to now, Jonkers *et al.* [83] used the detection of CaCO_3_ crystals at the crack surface as an indication of crack healing. However, it has not been investigated whether crystals also deposit inside the cracks. Wang *et al.* [118] added nitrate to the nutrients as an alternative electron acceptor to make bacterial CaCO_3_ precipitation possible without the presence of O_2_.

### 5.2. Exertion of Heat

When heat needs to be applied externally, the mechanism cannot be fully considered as an autonomous crack healing mechanism. Besides, heat supply raises the costs due to the work of heating and due to the need for inspection to find out whether cracks occurred. The need for inspection is circumvented in the approach of Nishiwaki *et al.* [129], at least when permanent electric current is supplied. However, both use of the strain sensitive sensor and continuous supply of current raise the cost of this system, and therefore, make it less convenient.

If overheated, concrete may deteriorate. Water in concrete is vaporized and diffused at temperatures over 100 °C, and dehydration and collapse of the microstructure occur at temperatures over 180 °C [148]. Isaacs *et al.* [44] heat until 90 °C to activate the PET shrinkable polymers, so no or limited damage will occur in the matrix. However, Dry [92] heats until 100 °C and Yuan *et al.* [89] even heat until 150 °C to reduce the viscosity of the melted EVA particles. These temperatures may already result in damage to the concrete matrix.

On the other hand, using heat to trigger the healing mechanism gives the advantage that the used encapsulation materials do not need to be brittle and thus casting of the specimens may become less difficult.

### 5.3. Crack Formation

The big advantage of using crack formation as the trigger mechanism is that the system responds very fast. Crack formation is immediately followed by release of the healing agent into the crack and healing only depends on curing of the agent.

To be sure that cracks break the capsules, good adhesion between the outer part of the capsule and the matrix is needed and capsule walls need to be preferentially brittle. However, the brittleness of the capsules makes it almost impossible to mix them into the concrete matrix. Therefore, an additional step may be needed upon concrete casting to place the capsules in the moulds. Another option would be to use encapsulation materials which are initially flexible and thus able to be mixed in and become more brittle over time to break when cracks run through as stated by Dry [138]. However, no research has focused on this topic up to now.

## 6. Regained Properties and Assessment of Healing Performance

In the subsequent paragraphs, the properties which can be regained due to self-healing are described. In Table 4 an overview of the test methods used to assess the healing efficiency, is given.

### 6.1. Increased Durability

The durability can be increased when self-healing of cracks results in retrieval of gas and water tightness. Several of the approaches aim at making concrete watertight; for example, when SAP particles are embedded inside the cementitious matrix. However, it should be noted that the swelling effect of SAP particles depends upon the type of liquid. Lee *et al.* [49] noticed complete sealing when tap water, synthetic groundwater or sodium chloride solution entered into the crack while swelling of the SAP particles was limited when synthetic seawater intruded. Therefore, they concluded that this self-healing approach is unlikely to be applicable in marine structures. Besides, upon freezing, water inside the saturated SAP particles will expand and cause damage due to the development of internal stresses. Furthermore, SAP particles will release their entrapped liquid and shrink during dry periods. This may cause concrete degradation as the released liquid may contain sulfate, chloride, *etc.* ions. In addition, SAP particles no longer form a barrier when they are not swollen. Moreover, SAP are not able to prevent the ingress of harmful gasses such as CO_2_ and O_2_ which may cause carbonation of the matrix and corrosion of the reinforcement.

Gas tightness is regained and water tightness becomes permanent and independent on the type of intruding liquid when the crack is filled with deposited crystals. In the system of Ahn and Kishi [78], Morimoto *et al.* [64] and Sisomphon and Copuroglu [70], cracks were completely filled with the deposited materials and became air and watertight.

Also cracks in the bacterial self-healing concrete proposed by Jonkers *et al.* [83] and Wang *et al.* [110], became gas and watertight after activation of the bacteria, consumption of the nutrients and crack filling with deposited CaCO_3_ crystals.

Cailleux and Pollet [1] and Van Tittelboom *et al.* [116] noted that the water permeability of damaged specimens containing capsules filled with polymeric healing agent was similar to values obtained for undamaged specimens. Yang *et al.* [115] measured the air permeability of specimens containing oil core/silica gel shell microcapsules and noticed that, relative to control samples, the highest percentage of decrease in permeability coefficient was observed for damaged self-healing mortar, *i.e.*, 50.2%.

Also for a real application, it was shown that encapsulated healing agent can make cracked structures watertight. Dry [93] created control joints on the surface of a concrete bridge deck as a transverse row of sealant filled tubes. These tubes were weaker than the concrete in tension so they broke due to shrinkage strains thereby focusing the transverse cracks along this line. An adhesive was then released from the tubes and sealed the cracks. The repair agent used had a low modulus thereby allowing further crack movement to maintain water tightness.

**Table 4:** Techniques used to evaluate the healing efficiency.

Technique		Possibilities	References
visualization and determination	Optical microscopy + image analysis	Visualization crystal deposition + determination healing rate	[38,39,70,78,103,105,107,110,149]
	Scanning electron microscopy	Visualization crystal deposition	[1,25,38,78,107,110,137]
	Environmental scanning electron microscopy	Visualization breakage of partially embedded capsule	[32]
	Thin section analysis	Visualization crystal deposition inside crack	[31,127,135]
	X-ray radiography	Visualization release encapsulated agent from embedded capsule	[111]
	X-ray tomography	Visualization release encapsulated agent from embedded capsule in 3D	[116]
	Digital image correlation	Visualization of crack closure upon heat treatment of SMA	[48]
	X-ray diffraction analysis	Determination of crystalline materials	[63,78]
	Ramann spectroscopy	Determination chemical composition	[38]
	Infrared analysis	Determination of precipitated products	[90,105]
regain tightness	Water permeability | low pressure	Water flow through (healed) crack	[1,14,15,38,39,40,64,70,79,129]
	Water permeability | high pressure	Water flow through (healed) crack	[16,22,103,116,118]
	Air permeability	Air flow through (healed) crack	[64,115]
	Capillary water uptake	Capillary water uptake by (healed) crack	[35,135]
	Neutron radiography	Visualize capillary water uptake by (healed) crack	[150,151]
	Corrosion test	Resistance against corrosion	[109,110,136]
	Frost salt scaling	Resistance against frost salt scaling	[152]
	Chloride diffusion	Resistance against chloride ingress	[50,60]
	Osmotic pressure	Resistance against ion ingress	[153]
	Ultrasonic transmission measurements	Continuity of material	[63]
regain mechanical properties	Compression test		[115,154]
	Tensile test	Regain in strength, stiffness and/or energy obtained when reloading healed specimen	[36,38,39,155]
	3-point bending test		[5,23,28,43,47,97,107,109,112,114,122]
	4-point bending test	Formation of new cracks *versus* reopening of old cracks	[96,100,116,147,156]
	Horizontal deformation column/frame		[100,157]
	Impact loading slab		[100]
	Acoustic emission analysis	Regain in energy/Notice capsule breakage	[13,158,159]
	Resonance frequency analysis	Regain in stiffness	[11,13,16,17,36,85,153]

### 6.2. Regain in Mechanical Properties

Although, regaining air and water tightness is the most important objective of self-healing concrete, many researchers have focused on the possibility of regaining mechanical properties due to autonomous crack healing.

When cracks are repaired due to autogenous healing or improved autogenous healing, the mechanical properties after healing, will mostly be inferior compared to virgin specimens. Although, the materials which fill the crack (secondary hydration products and CaCO_3_ crystals) are identical to the constituting elements of the cementitious matrix, CSH gel formed due to ongoing hydration seems to have inferior mechanical properties compared to primary hydration products, and deposited CaCO_3_ crystals do not form a proper bond with the crack faces. This implies that also by using bacterial CaCO_3_ precipitation, mechanical properties of the virgin material will never be regained completely.

Only in the case when polymeric based healing agents are encapsulated, more than 100% of regain in strength and stiffness can be obtained. Regain will depend on the type of capsules and subsequently the released volume of healing agent and the type of healing agent. While strength regain will be limited for foam like materials and silicones, epoxy resins and CA can result in more than 100% strength regain.

## 7. Future Perspectives

From the moment S. White published his paper in Nature, the interest in self-healing materials increased rapidly. To obtain self-healing properties in cementitious materials, different approaches have been proposed by research groups all over the world, as shown in this review. However, it is quite difficult to select the most efficient approach as each research group uses its own test methods to evaluate the healing efficiency. In our opinion, the development of standard test methods would be very useful to compare the efficiency of one approach against another and should thus be one of the future goals.

While it is not yet possible to predict in detail which technique would be better than the other one, we can already give our general opinion about the effectiveness of the different approaches. In the past, a lot of research was devoted to autogenous healing, but for two reasons we would suggest to focus future research on autonomous healing (capsule and vascular based self-healing approaches). The first reason is that autogenous healing will always remain restricted to small cracks and the second reason is that the reliability of autogenous healing is lower as it will always depends on the composition of the matrix, determining the feasible reaction mechanisms, at the moment of crack formation.

With regard to capsule- and tubular based self-healing, healing agents which react due to contact with moisture or air, seem less interesting as premature curing of the agent inside the capsules may occur and the reaction will not start immediately after release. Also reaction upon heating is less interesting as human intervention is still needed and this will raise the cost. According to the opinion of the authors, reaction with (a second component present in) the cementitious matrix or a second component provided by additional capsules is most interesting.

Apart from the demands related to curing of the healing agent, other requirements depend on the intended application. Do you only cope with early age cracks, what is the expected crack width, does the width remain stable or are you dealing with dynamic cracks, does water flow into the crack, is strength regain needed in addition to recovery of the air- and liquid tightness, does the aesthetic view matter, *etc.*?

To make the capsule based approach practically applicable, it is important that future research is devoted to the development of capsules which are able to survive the concrete mixing and manufacturing process while not influencing the mechanical properties of the concrete too much. Tubular or spherical capsules may be used depending on what is the most efficient to respond to the before mentioned properties and taken into account that the probability of a crack hitting a capsule, the release efficiency and the healable crack volume should be high enough. The tubular encapsulation approach will only be applicable for precast concrete elements as manual introduction of tubular capsules is needed. The advantage of this system is that the chance of a crack hitting the tubes is higher and that the tubes can eventually be replenished if needed.

It should become clear from this review paper that self-healing concrete is a truly interdisciplinary research topic involving microbiology, chemistry, material science, civil engineering, *etc.* In order to develop a practically applicable self-healing approach cross-disciplinary cooperation and communication between researchers with different expertise will be of utmost importance.

However, the *in situ* application of any self-healing approach will mainly depend on the required costs. Therefore, it is of utmost importance to keep the costs of the mechanism as low as possible and preferentially lower than the sum of direct and indirect repair costs which appear over the lifetime of the structure. Only this may help to convince manufacturers to make use of self-healing concrete.
